# Supplementary material for: Similar yet different: phylogenomic analysis to delineate Salmonella and Citrobacter species boundaries
Source: BMC Genomics. 2020 May 29;21:377. doi: 10.1186/s12864-020-06780-y (PMC7257147; doi:10.1186/s12864-020-06780-y)
Supplement: Supplementary file 1 — Additional file 1: Table S1. Phenotypic characterization of Citrobacter and Salmonella strains. [file 12864_2020_6780_MOESM1_ESM.docx]

**Table S1.** Phenotypic characterization of *Citrobacter* and *Salmonella* strains^a,b^

| Isolate | Strain/Serovar Name | Latex test | API20E | | VITEK 2 | MALDI-TOF | |
| --- | --- | --- | --- | --- | --- | --- | --- |
|  |  |  | **Score** | **Result** |  | **Score** | **Identity** |
| S646 | *Citrobacte*r sp. | positive | 1644573 | *C. freundii* 90.8% | Unidentified | 2.08 | *C. farmeri* |
| S647 | *Citrobacte*r sp | positive | 1644573 | *C. freundii* 90.8% | Unidentified | 2.08 | *C. farmeri* |
| S648 | *Citrobacte*r sp | positive | 1644573 | *C. freundii* 90.8% | Unidentified | 2.08 | *C. farmeri* |
| S1284 | *Citrobacte*r sp | positive | 1644553 | *C. braakii* 74.4% | *C. sedlakii* 86% | 2.46 | *C. braakii* |
| S1285 | *Citrobacte*r sp | positive | 1644573 | *C. freundii* 90.8% | Unidentified | 2.22 | *C. farmeri* |
| S63 | *C. amalonaticus* | ND^c^ | 3744573 | *C. braakii* 87.6% | ND | 2.00 | *C. farmeri* |
| S62 | *C. braakii* | ND | 3704553 | *C. braakii* 99.7% | ND | 2.23 | *C. braakii* |
| S61 | *C. freundii* | ND | 1744553 | *C. braakii* 99.7% | ND | 2.29 | *C. freundii* |
| S79 | *C. koseri* | ND | 1344513 | *C. braakii* 99.2% | ND | 2.22 | *C. koseri* |
| S78 | *C. werkmanii* | ND | 3604512 | *C. youngae* 99.8% | ND | 2.29 | *C. freundii* |
| S187 | Enteritidis | positive | 6704552 | *Salmonella* spp*.* 89.6% | ND | 2.31 | *Salmonella* spp. |
| S437 | Typhimurium | ND | 6704752 | *Salmonella* spp*.* 99.8% | ND | 2.19 | *Salmonella* spp. |
| S191 | Heidelberg | ND | 6704552 | *Salmonella* spp*.* 89.6% | ND | 2.21 | *Salmonella* spp. |
| S35 | Dusseldorf | ND | 6704752 | *Salmonella* spp*.* 99.8% | ND | 2.06 | *Salmonella* spp. |
| S341 | Daytona | ND | 4704712 | *Salmonella* spp*.* 99.7% | ND | 2.16 | *Salmonella* spp. |
| S25 | Amager | ND | 6704712 | *Salmonella* spp*.* 99.5% | ND | 1.95 | *Salmonella* spp. |

^a^ For reference the API for *E*. *coli* ATCC25922 is 5144572

^b^ *S*. *enterica* strains are listed by serovar name

^c^ ND = not done
