# Supplementary material for: Similar yet different: phylogenomic analysis to delineate Salmonella and Citrobacter species boundaries
Source: BMC Genomics. 2020 May 29;21:377. doi: 10.1186/s12864-020-06780-y (PMC7257147; doi:10.1186/s12864-020-06780-y)
Supplement: Supplementary file 2 — Additional file 2: Table S2. List of reference genomes used in this study. [file 12864_2020_6780_MOESM2_ESM.docx]

**Table S2.** List of reference genomes used in this study.

| Organism | Accession number |
| --- | --- |
| *Citrobacter amalonaticus* FDAARGOS 165 | NZ_CP014070.1 |
| *Citrobacter amalonaticus* Y19 | NZ_CP011132. |
| *Citrobacter braakii* GTA CB04 | JRHL01000001.1 |
| *Citrobacter farmeri* GTC 1319 | BBMX01000001.1 |
| *Citrobacter freundii* ATCC 8090 | ANAV01000001.1 |
| *Citrobacter freundii* CFNIH1 | NZ_CP007557.1 |
| *Citrobacter koseri* ATCC BAA895 | NC_009792.1 |
| *Citrobacter rodentium* ICC168 | NC_013716.1 |
| *Citrobacter sedlakii* NBRC 105722 | BBNB01000001.1 |
| *Citrobacter werkmanii* NBRC 105721 | BBMW01000001.1 |
| *Citrobacter youngae* ATCC 29220 | NZ_ABWL02000000 |
| *Cronobacter sakazakii* ATCC 29544 | NZ_CP011047.1 |
| *Escherichia albertii* KF1 | NZ_CP007025.1 |
| *Escherichia coli* K-12 substr. MG1655 | NC_000913.3 |
| *Escherichia coli* O157:H7 Sakai | NC_002695.1 |
| *Escherichia fergusonii* ATCC 35469 | NC_011740.1 |
| *Enterobacter aerogenes* KCTC 2190 | NC_015663.1 |
| *Enterobacter cloacae* subsp. *cloacae* ATCC 13047 | NC_014121.1 |
| *Erwinia amylovora* CFBP1430 | NC_013961.1 |
| *Hafnia alvei* FB1 | NZ_CP009706.1 |
| *Hafnia paralvei* ATCC 29927 | NZ_LXET01000001.1 |
| *Kluyvera intermedia* CAV1151 | NZ_CP011602.1 |
| *Klebsiella pneumoniae* subsp. *pneumoniae* HS11286 | NC_016845.1 |
| *Morganella morganii* subsp. *morganii* KT | NC_020418.1 |
| *Pantoea agglomerans* C410P1 | NZ_CP016889.1 |
| *Pantoea dispersa* EGD-AAK13 | NZ_AVSS01000001.1 |
| *Proteus mirabilis* HI4320 | NC_010554.1 |
| *Proteus vulgaris* CYPV1 | NZ_CP012675.1 |
| *Salmonella bongori NCTC 12419* | FR877557.1 |
| *Salmonella enterica* subsp. *arizonae* serovar 62:z36:- RKS2983 | NZ_CP006693.1 |
| *Salmonella enterica* subsp. *diarizonae* 11-01853 | NZ_CP011289.1 |
| *Salmonella enterica* subsp. *houtenae* ATCC BAA-1581 | AGRM01000001.1 |
| *Salmonella enterica* subsp. *enterica* ser. Enteritidis P125109 | NC_011294.1 |
| *Salmonella enterica* subsp*. enterica* ser. Gallinarum 287/91 | NC_011274.1 |
| *Salmonella enterica* subsp. *enterica* ser. Paratyphi A AKU12601 | FM200053.1 |
| *Salmonella enterica* subsp*. enterica* ser.Typhi CT18 | NC_003198.1 |
| *Salmonella enterica* subsp*. enterica* ser.Typhimurium LT2 | AE006468.2 |
| *Serratia marcescens* subsp. *marcescens* Db11 | NZ_HG326223.1 |
| *Shigella dysenteriae* Sd197 | NC_007606.1 |
| *Shigella flexneri* 2a str. 301 | NC_004337.2 |
| *Sodalis glossinidius* str. morsitans | NC_007712.1 |
| *Yersinia enterocolitica* subsp. *enterocolitica* 8081 | NC_008800.1 |
