# Supplementary material for: Similar yet different: phylogenomic analysis to delineate Salmonella and Citrobacter species boundaries
Source: BMC Genomics. 2020 May 29;21:377. doi: 10.1186/s12864-020-06780-y (PMC7257147; doi:10.1186/s12864-020-06780-y)
Supplement: Supplementary file 4 — Additional file 4: Table S4. Number of open reading frames (ORFS) and coding sequences (CDS) in the genomes of SalFoS strains. [file 12864_2020_6780_MOESM4_ESM.docx]

**Table S4. Number of open reading frames (ORFS) and coding sequences (CDS) in the genomes of SalFoS strains.**

| Strains | **ORFS** | **CDS** |
| --- | --- | --- |
| Amager S25 | 4588 | 4496 |
| Anatum S234 | 4420 | 4326 |
| Braenderup S208 | 4601 | 4514 |
| Brandenburg S291 | 4419 | 4326 |
| *C. amalonaticus* S63 | 5285 | 5195 |
| *C. amalonaticus* S68 | 4701 | 4608 |
| *C. braakii* S58 | 4619 | 4531 |
| *C. braakii* S64 | 4931 | 4838 |
| *C. braakii* S85 | 4873 | 4785 |
| *C. braakii* S86 | 4825 | 4735 |
| *C. farmeri* S80 | 4925 | 4832 |
| *C. freundii* S20 | 5075 | 4981 |
| *C. freundii* S61 | 4543 | 4456 |
| *C. freundii* S66 | 4782 | 4683 |
| *C. freundii* S67 | 4967 | 4877 |
| *C. freundii* S83 | 5068 | 4977 |
| *C. freundii* S89 | 4694 | 4603 |
| *C. koseri* S65 | 4619 | 4524 |
| *C. koseri* S79 | 4699 | 4605 |
| *C. werkmanii* S78 | 5366 | 5262 |
| Chingola S32 | 4478 | 4388 |
| *Citrobacter* sp. S1278 | 4903 | 4818 |
| *Citrobacter* sp. S1279 | 4903 | 4818 |
| *Citrobacter* sp. S1280 | 4898 | 4813 |
| *Citrobacter* sp. S1281 | 4897 | 4812 |
| *Citrobacter* sp. S1282 | 5043 | 4956 |
| *Citrobacter* sp. S1283 | 4906 | 4821 |
| *Citrobacter* sp. S1284 | 5245 | 5152 |
| *Citrobacter* sp. S1285 | 4767 | 4683 |
| *Citrobacter* sp. S60 | 4710 | 4619 |
| *Citrobacter* sp. S646 | 5249 | 5159 |
| *Citrobacter* sp. S647 | 5270 | 5180 |
| *Citrobacter* sp. S648 | 5223 | 5133 |
| *Citrobacter* sp. S649 | 5227 | 5137 |
| *Citrobacter* sp. S77 | 4609 | 4514 |
| *Citrobacter* sp. S81 | 5092 | 4992 |
| *Citrobacter* sp. S91 | 5246 | 5157 |
| *Citrobacter* sp. S93 | 5356 | 5269 |
| *Citrobacter* sp. S94 | 4622 | 4534 |
| *Citrobacter* sp. S95 | 4790 | 4697 |
| *Citrobacter* sp. S96 | 4719 | 4631 |
| *Citrobacter* sp. S97 | 4725 | 4639 |
| Daytona S341 | 4496 | 4408 |
| Derby S228 | 4594 | 4500 |
| Duesseldorf S35 | 4473 | 4381 |
| Elisabethville S36 | 4846 | 4756 |
| Enteritidis S186 | 4518 | 4426 |
| Enteritidis S187 | 4540 | 4449 |
| Enteritidis S420 | 4515 | 4417 |
| Enteritidis S422 | 4561 | 4462 |
| Enteritidis S424 | 4669 | 4576 |
| Fresno S38 | 4413 | 4323 |
| Gallinarum S173 | 4624 | 4541 |
| Hadar S219 | 4440 | 4348 |
| Heidelberg S190 | 4537 | 4449 |
| Heidelberg S191 | 4536 | 4448 |
| Heidelberg S370 | 4663 | 4565 |
| Heidelberg S431 | 4659 | 4569 |
| Indikan S41 | 4476 | 4382 |
| Infantis S198 | 4336 | 4240 |
| Javiana S200 | 4567 | 4471 |
| Javiana S201 | 4460 | 4368 |
| Javiana S202 | 4396 | 4307 |
| Javiana S203 | 4361 | 4269 |
| Kentucky S245 | 4932 | 4836 |
| Litchfield S272 | 4588 | 4493 |
| Luciana S43 | 4363 | 4277 |
| Montevideo S239 | 5403 | 5302 |
| Newport S195 | 4575 | 4481 |
| Newport S196 | 4648 | 4555 |
| Newport S197 | 4467 | 4375 |
| Newport S2 | 4561 | 4471 |
| Newport S566 | 4351 | 4257 |
| Ohio S315 | 4449 | 4356 |
| Oranienburg S216 | 4371 | 4278 |
| Orientalis S45 | 4400 | 4308 |
| Paratyphi A S399 | 4480 | 4392 |
| Pasing S46 | 4402 | 4309 |
| Poona S307 | 4361 | 4268 |
| Saintpaul S204 | 4455 | 4363 |
| Senftenberg S271 | 4726 | 4630 |
| Solt S47 | 4368 | 4277 |
| Tado S48 | 4465 | 4374 |
| Taiping S49 | 4565 | 4476 |
| Tennessee S343 | 4682 | 4589 |
| Typhi S404 | 4405 | 4317 |
| Typhimurium S188 | 4590 | 4497 |
| Typhimurium S189 | 4619 | 4524 |
| Typhimurium S373 | 4612 | 4515 |
| Typhimurium S415 | 4874 | 4781 |
| Typhimurium S418 | 4824 | 4731 |
| Typhimurium S437 | 4708 | 4611 |
| Typhimurium S441 | 4661 | 4570 |
| Tyresoe S51 | 4419 | 4331 |
| Weston S54 | 4376 | 4288 |
|  |  |  |
