# Supplementary material for: Similar yet different: phylogenomic analysis to delineate Salmonella and Citrobacter species boundaries
Source: BMC Genomics. 2020 May 29;21:377. doi: 10.1186/s12864-020-06780-y (PMC7257147; doi:10.1186/s12864-020-06780-y)
Supplement: Supplementary file 6 — Additional file 6: Table S6. List of Citrobacter genomes obtained from RefSeq database, June 2019. [file 12864_2020_6780_MOESM6_ESM.docx]

**Table S6. List of *Citrobacter* genomes obtained from RefSeq database, June 2019**

| **Strain** | **RefSeq assembly accession** |
| --- | --- |
| *C. amalonaticus* | GCF00731055 |
| *C. amalonaticus* | GCF00972645 |
| *C. amalonaticus* | GCF00981805 |
| *C. amalonaticus* | GCF01276125 |
| *C. amalonaticus* | GCF01373155 |
| *C. amalonaticus* | GCF01471655 |
| *C. amalonaticus* | GCF01558935 |
| *C. amalonaticus* | GCF01559075 |
| *C. amalonaticus* | GCF02918535 |
| *C. amalonaticus* | GCF02918555 |
| *C. amalonaticus* | GCF02918935 |
| *C. amalonaticus* | GCF02919495 |
| *C. amalonaticus* | GCF03938795 |
| *C. amalonaticus* | GCF04167485 |
| *C. braakii* | GCF00786265 |
| *C. braakii* | GCF00786275 |
| *C. braakii* | GCF01022685 |
| *C. braakii* | GCF01059745 |
| *C. braakii* | GCF01062165 |
| *C. braakii* | GCF01065805 |
| *C. braakii* | GCF01067775 |
| *C. braakii* | GCF01952715 |
| *C. braakii* | GCF02073755 |
| *C. braakii* | GCF02075345 |
| *C. braakii* | GCF02208845 |
| *C. braakii* | GCF02239605 |
| *C. braakii* | GCF02918455 |
| *C. braakii* | GCF02918465 |
| *C. braakii* | GCF02918495 |
| *C. braakii* | GCF02918575 |
| *C. braakii* | GCF02919425 |
| *C. braakii* | GCF02919455 |
| *C. braakii* | GCF02919485 |
| *C. braakii* | GCF02923765 |
| *C. braakii* | GCF02939255 |
| *C. braakii* | GCF04331445 |
| *C. braakii* | GCF04331535 |
| *C. braakii* | GCF04331575 |
| *C. braakii* | GCF04331585 |
| *C. braakii* | GCF04331635 |
| *C. europaeus* | GCF03795375 |
| *C. europaeus* | GCF900079995 |
| *C. europaeus* | GCF900080005 |
| *C. farmeri* | GCF00764735 |
| *C. farmeri* | GCF02249995 |
| *C. farmeri* | GCF03938205 |
| *C. freundii* | GCF00208765 |
| *C. freundii* | GCF00388155 |
| *C. freundii* | GCF00648515 |
| *C. freundii* | GCF00692115 |
| *C. freundii* | GCF00714305 |
| *C. freundii* | GCF00734905 |
| *C. freundii* | GCF00783995 |
| *C. freundii* | GCF00937505 |
| *C. freundii* | GCF01057215 |
| *C. freundii* | GCF01058505 |
| *C. freundii* | GCF01058675 |
| *C. freundii* | GCF01273815 |
| *C. freundii* | GCF01316675 |
| *C. freundii* | GCF01317155 |
| *C. freundii* | GCF01412725 |
| *C. freundii* | GCF01482545 |
| *C. freundii* | GCF01689745 |
| *C. freundii* | GCF01718055 |
| *C. freundii* | GCF01880795 |
| *C. freundii* | GCF01880845 |
| *C. freundii* | GCF01880865 |
| *C. freundii* | GCF01880945 |
| *C. freundii* | GCF01922445 |
| *C. freundii* | GCF02151735 |
| *C. freundii* | GCF02151755 |
| *C. freundii* | GCF02151785 |
| *C. freundii* | GCF02151815 |
| *C. freundii* | GCF02189125 |
| *C. freundii* | GCF02211705 |
| *C. freundii* | GCF02252125 |
| *C. freundii* | GCF02417535 |
| *C. freundii* | GCF02786865 |
| *C. freundii* | GCF02863945 |
| *C. freundii* | GCF02864025 |
| *C. freundii* | GCF02871775 |
| *C. freundii* | GCF02880615 |
| *C. freundii* | GCF02903215 |
| *C. freundii* | GCF02903305 |
| *C. freundii* | GCF02918505 |
| *C. freundii* | GCF02918835 |
| *C. freundii* | GCF02918865 |
| *C. freundii* | GCF02919795 |
| *C. freundii* | GCF02919825 |
| *C. freundii* | GCF03015305 |
| *C. freundii* | GCF03019835 |
| *C. freundii* | GCF03114935 |
| *C. freundii* | GCF03175795 |
| *C. freundii* | GCF03195445 |
| *C. freundii* | GCF03362775 |
| *C. freundii* | GCF03363295 |
| *C. freundii* | GCF03400395 |
| *C. freundii* | GCF03665595 |
| *C. freundii* | GCF03665615 |
| *C. freundii* | GCF03665655 |
| *C. freundii* | GCF03937345 |
| *C. freundii* | GCF04004965 |
| *C. freundii* | GCF04005085 |
| *C. freundii* | GCF04023995 |
| *C. freundii* | GCF04024405 |
| *C. freundii* | GCF04103775 |
| *C. freundii* | GCF04145665 |
| *C. freundii* | GCF04305705 |
| *C. freundii* | GCF04327825 |
| *C. freundii* | GCF900169625 |
| *C. freundii* | GCF900169695 |
| *C. freundii* | GCF900446835 |
| *C. freundii* | GCF900446875 |
| *C. freundii* | GCF900520375 |
| *C. gillenii* | GCF03429605 |
| *C. koseri* | GCF01057775 |
| *C. koseri* | GCF01471775 |
| *C. koseri* | GCF01546305 |
| *C. koseri* | GCF01546325 |
| *C. koseri* | GCF01552875 |
| *C. koseri* | GCF02393245 |
| *C. koseri* | GCF02863965 |
| *C. koseri* | GCF03184045 |
| *C. koseri* | GCF03226155 |
| *C. koseri* | GCF03350185 |
| *C. koseri* | GCF03938785 |
| *C. koseri* | GCF04181905 |
| *C. koseri* | GCF04181995 |
| *C. koseri* | GCF900446895 |
| *C. koseri* | GCF900446955 |
| *C. koseri* | GCF900446975 |
| *C. koseri* | GCF900460985 |
| *C. koseri* | GCF900461045 |
| *C. koseri* | GCF900461055 |
| *C. koseri* | GCF900461175 |
| *C. koseri* | GCF900706735 |
| *C. pasteurii* | GCF00826205 |
| *C. pasteurii* | GCF03665575 |
| *C. portucalensis* | GCF00238735 |
| *C. portucalensis* | GCF00521945 |
| *C. portucalensis* | GCF01281005 |
| *C. portucalensis* | GCF02042885 |
| *C. portucalensis* | GCF02051555 |
| *C. portucalensis* | GCF02843195 |
| *C. portucalensis* | GCF03471655 |
| *C. portucalensis* | GCF03990165 |
| *C. rodentium* | GCF00759815 |
| *C. rodentium* | GCF00835925 |
| *C. sedlakii* | GCF00759835 |
| *C. werkmanii* | GCF00759755 |
| *C. werkmanii* | GCF02025225 |
| *C. werkmanii* | GCF02099305 |
| *C. werkmanii* | GCF02185305 |
| *C. werkmanii* | GCF02386385 |
| *C. werkmanii* | GCF03665555 |
| *C. werkmanii* | GCF04146135 |
| *C. youngae* | GCF00155975 |
| *C. youngae* | GCF03255895 |
| *C. youngae* | GCF03818115 |
| *C. youngae* | GCF900446905 |
