# Supplementary material for: Similar yet different: phylogenomic analysis to delineate Salmonella and Citrobacter species boundaries
Source: BMC Genomics. 2020 May 29;21:377. doi: 10.1186/s12864-020-06780-y (PMC7257147; doi:10.1186/s12864-020-06780-y)
Supplement: Supplementary file 8 — Additional file 8. Supplementary information. [file 12864_2020_6780_MOESM8_ESM.zip › SI_9Nov2019.docx]

**SUPPLEMENTARY INFORMATION**

**METHODS**

**rRNA sequence analysis.** 16S rRNA analysis was performed to assess the phylogenetic relationship between *Citrobacter* spp., *S. enterica,* and members of Family *Enterobacteriaceae*. The 16S rRNA sequences were extracted from genome sequences using RNAmmer version 1.2 (1) and aligned using MUSCLE v3.8.31 (2). Maximum likelihood phylogenetic trees were constructed using RAxML version 8.2.9 (3) with 1,000 bootstrap replications for nodal support.

**Average nucleotide identity.** As a measure of genetic relatedness and to determine whether a given pair of genomes belong to the same species, the average nucleotide identity (ANI) values based on pairwise whole genome comparisons were determined between the false positive strains and representative *Citrobacter*, *S. enterica,* and *E. coli* genome sequences using JSpeciesWS at <http://jspecies.ribohost.com/jspeciesws/> (4).

**REFERENCES**

1. **Lagesen K, Hallin P, Rodland EA, Staerfeldt HH, Rognes T, Ussery DW.** 2007. RNAmmer: consistent and rapid annotation of ribosomal RNA genes. Nucleic Acids Res **35:**3100-3108.

2. **Edgar RC.** 2004. MUSCLE: multiple sequence alignment with high accuracy and high throughput. Nucleic Acids Res **32:**1792-1797.

3. **Stamatakis A.** 2014. RAxML version 8: a tool for phylogenetic analysis and post-analysis of large phylogenies. Bioinformatics **30:**1312-1313.

4. **Richter M, Rossello-Mora R, Oliver Glockner F, Peplies J.** 2016. JSpeciesWS: a web server for prokaryotic species circumscription based on pairwise genome comparison. Bioinformatics **32:**929-931.

**FIGURE LEGEND**

**Figure S1. Phylogenetic tree based on 16S rRNA sequence analysis.** A consensus tree based on the alignment of 16S rRNA sequences of the *Citrobacter* produce isolates falsely identified as *Salmonella* and members of the family *Enterobacteriaceae* provided limited resolution. Genetic distance is defined by the scale and bootstrap values indicate percentages of 1,000 replicates. False positive strains except for *Citrobacter* sp. S1284 are grouped together in Group 3: S646, S647, S648, S1285. Other *Citrobacter* strains were also included in the analysis and clustered in the collapsed nodes as Group 1 - S1278, S1279, S1281, S649 and Group 2 - S1280, S1282, S1283.
