# Supplementary figures and images for: Similar yet different: phylogenomic analysis to delineate Salmonella and Citrobacter species boundaries
Source: BMC Genomics. 2020 May 29;21:377. doi: 10.1186/s12864-020-06780-y (PMC7257147; doi:10.1186/s12864-020-06780-y)

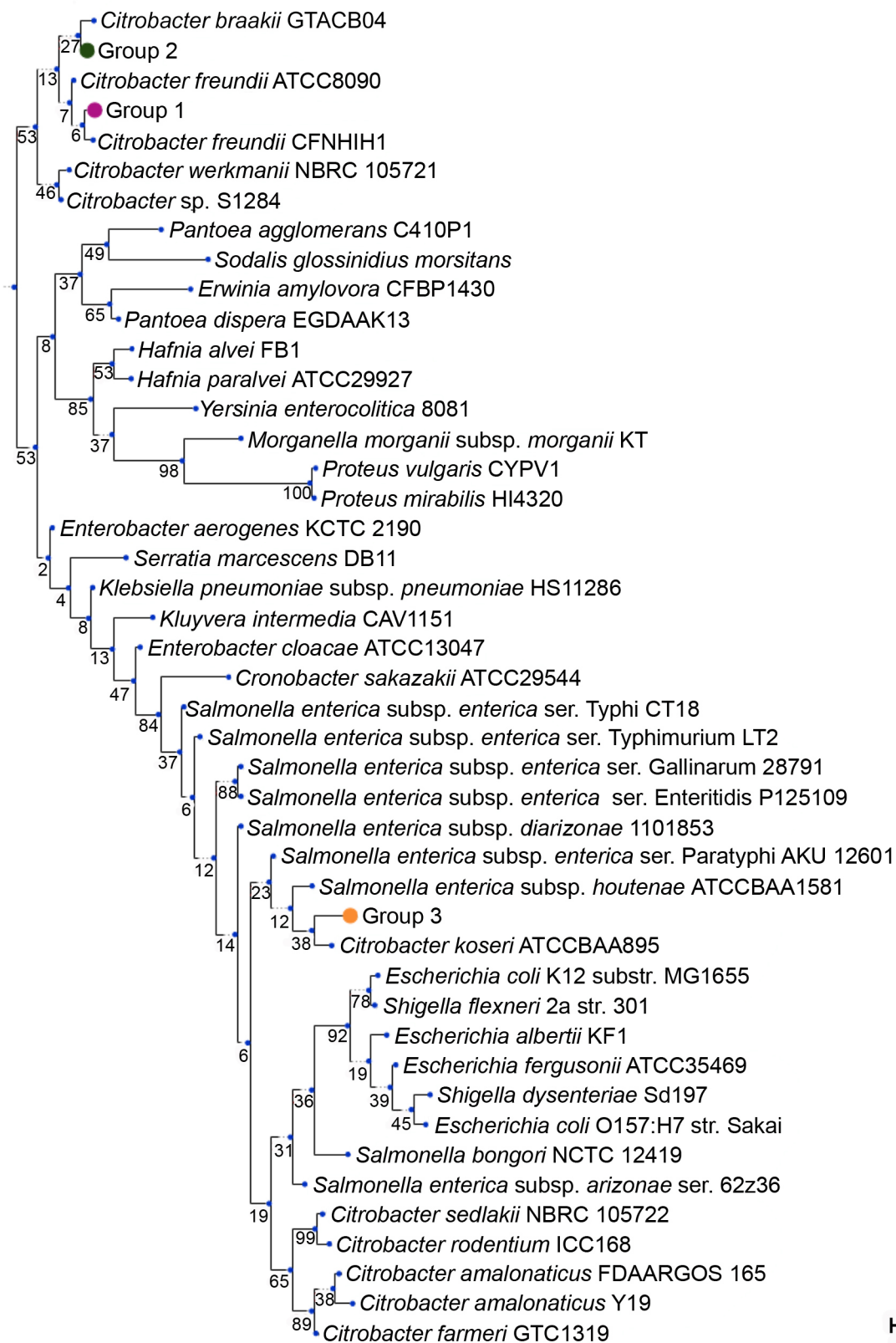

Supplement: Supplementary file 8 — Additional file 8. Supplementary information. [file 12864_2020_6780_MOESM8_ESM.zip › FigureS1_16s_1oct2018.pdf]
